# Emerging applications of artificial intelligence for risk stratification in head and neck cancer: a scoping review

**DOI:** 10.3389/fonc.2026.1803391

**Published:** 2026-05-28

**Authors:** Valeria Concha Fernández, Mariana González Garcés, Jerónimo Cárdenas Montoya, Mario Andrés Torres Torres, Erwin Hernando Hernández Rincón

**Affiliations:** 1 School of Medicine, Universidad de La Sabana, Chía, Colombia; 2 Department of Family Medicine and Public Health, Universidad de La Sabana, Chía, Colombia

**Keywords:** artificial intelligence, clinical decision support, deep learning, extranodal extension, head and neck cancer, lymph node metastasis, machine learning, precision oncology

## Abstract

**Introduction:**

Head and neck cancer represents a major clinical challenge due to its pronounced biological, histopathological and anatomical heterogeneity, which limits the predictive accuracy of conventional staging systems. To optimize diagnostic and therapeutic decision-making, reduce overtreatment and advance towards more precise oncology, robust risk stratification is essential. In recent years, artificial intelligence (AI) has emerged as a promising tool to support these processes through advanced analysis of clinical, radiological and histopathological data.

**Objective:**

To identify and describe the available scientific evidence on emerging applications of AI in risk stratification for head and neck cancer.

**Methods:**

A scoping review was conducted in accordance with the methodological guidance of the Joanna Briggs Institute and the recommendations of the PRISMA-ScR checklist. Studies published between January 2015 and January 2026, in English or Spanish, were identified through systematic searches of PubMed, Scopus, Web of Science and IEEE Xplore, supplemented by manual reference screening. Study selection, data extraction and evidence synthesis were performed independently by two reviewers using the Population–Concept–Context (PCC) framework.

**Results:**

A total of 44 studies were included, applying AI techniques primarily to diagnostic tasks and prognostic risk stratification in head and neck cancer, including prediction of lymph node metastasis and extranodal extension. The most frequently employed approaches were machine learning models, deep learning architectures and radiomics-based methods. Commonly used data modalities included computed tomography, magnetic resonance imaging, digital histopathology and structured clinical variables. Overall, studies reported moderate to high predictive performance; however, the evidence was characterized by substantial methodological heterogeneity, a predominance of retrospective designs, limited external validation and insufficient assessment of the clinical impact of the proposed models.

**Conclusions:**

The available evidence suggests that AI has the potential to enhance risk stratification in head and neck cancer, complementing conventional clinical approaches and contributing to the development of more individualized oncology. Nevertheless, responsible clinical implementation of these technologies requires overcoming challenges related to methodological standardization, prospective multicentre validation, model interpretability, and the consideration of ethical and equity-related issues.

**Systematic review registration:**

https://osf.io/7aem4/overview.

## Introduction

Head and neck cancer, encompassing tumors of the oral cavity, oropharynx, hypopharynx and larynx, constitutes a major global public health problem due to its high morbidity and mortality, substantial functional impact and marked deterioration in quality of life, particularly in advanced stages (1, 2). Despite therapeutic advances in surgery, radiotherapy, chemotherapy and immunotherapy, overall survival remains limited and heterogeneous, underscoring the need for more precise strategies for early diagnosis and risk stratification (3, 4).

Predicting the clinical behavior of head and neck cancer represents a substantial challenge. Factors such as tumor biological heterogeneity, anatomical diversity, the etiological influence of human papillomavirus infection and inter-individual variability in treatment response limit the ability of conventional staging systems, including TNM, to adequately estimate individual patient risk (4–6). In this context, more accurate risk stratification is essential to optimize clinical decision-making and advance towards personalized medicine models.

In recent years, AI has emerged as a tool with transformative potential in oncology. Machine learning and deep learning models have demonstrated the ability to analyze large volumes of complex data, identify non-linear patterns and improve diagnostic and prognostic accuracy across diverse clinical scenarios (6–8). In head and neck cancer, these methodologies have been applied to support diagnosis, early detection, tumor classification and the prediction of clinically relevant outcomes such as survival, recurrence and nodal dissemination (6, 7).

One of the most extensively developed areas is the application of radiomics, which enables the extraction of quantitative features from conventional medical imaging modalities such as computed tomography, magnetic resonance imaging and PET/CT. Recent studies have shown that radiomics-based and deep learning approaches can capture latent tumor information related to biological aggressiveness, heterogeneity and progression risk, providing additional prognostic value beyond conventional visual assessment (6–9). These techniques have been employed to predict lymph node metastasis, extranodal extension and locoregional recurrence in head and neck cancer (8, 9).

Complementarily, AI has been applied to the analysis of digital histopathological images using whole-slide imaging, as well as to the integration of clinical and molecular data. These approaches have enabled refinement of tumor classification, identification of risk subgroups and support for therapeutic decision-making, consolidating multimodal strategies as one of the most promising emerging trends in precision oncology (10, 11).

Nevertheless, despite the rapid growth of the literature, the available evidence exhibits substantial methodological heterogeneity. Studies differ widely in terms of design, sample size, data sources, algorithms employed, performance metrics and validation strategies (11–13). A considerable proportion relies on retrospective cohorts and internal validation, raising important concerns regarding reproducibility, generalizability and clinical applicability of the proposed models (12, 13). In addition, challenges persist related to integration into clinical practice, the risk of algorithmic bias, workflow standardization and model interpretability (13, 14).

In this context, a structured synthesis is required not only to map emerging applications of AI, but also to systematically organize the clinical domains addressed, the methodological approaches employed and the levels of validation reported. A scoping review represents the most appropriate methodological design for this purpose, as it allows the integration of studies with heterogeneous objectives, designs and methods, distinguishes consolidated trends from exploratory approaches, and identifies knowledge gaps not addressed by previous narrative reviews (13, 15).

Therefore, the objective of this scoping review is to map and characterize the existing evidence on the use of AI for risk stratification in head and neck cancer, describing methodological approaches, data types, evaluated outcomes and the main reported limitations, in order to guide future research and promote responsible, evidence-based clinical implementation (12, 15).

## Methodology

### Study design

A scoping review was conducted with the aim of systematically mapping and describing the existing scientific evidence on emerging applications of AI in the diagnosis and risk stratification of head and neck cancer. This methodological design was selected for its suitability to synthesize heterogeneous evidence in terms of study designs, data sources, algorithmic approaches and clinical objectives, as well as to identify emerging trends and knowledge gaps within a rapidly evolving field.

The review was developed in accordance with the methodological guidance of the Joanna Briggs Institute (JBI) for scoping reviews and reported following the recommendations of the PRISMA-ScR checklist, in order to ensure transparency, reproducibility and methodological rigor throughout all stages of the process.

### Research question and PCC framework

The research question was structured using the Population–Concept–Context (PCC) framework to explicitly guide the identification, selection and synthesis of the included studies.

The population comprised adult patients with confirmed diagnoses or clinical suspicion of head and neck cancer, including tumors of the oral cavity, oropharynx, hypopharynx, larynx and other related anatomical structures.

The concept was defined as the application of AI techniques including machine learning algorithms, deep learning models, radiomics and hybrid approaches aimed at oncological diagnosis and risk stratification, such as prediction of lymph node metastasis, extranodal extension, tumor recurrence, survival or treatment response.

The context included clinical, hospital and academic settings in which real-world clinical data are used to support decision-making in head and neck cancer, encompassing medical imaging (CT, MRI, PET/CT), digital histopathological images and multimodal approaches integrating clinical, radiological and pathological information.

The research question was: What is the available evidence on the applications of AI in the diagnosis and risk stratification of head and neck cancer?

### Search strategy and data collection

A systematic bibliographic search was conducted across four electronic databases: PubMed, Scopus, Web of Science and IEEE Xplore, selected for their complementary coverage of biomedical, clinical and computational sciences.

Studies published in English or Spanish between January 2015 and January 2026 were included. This time frame was chosen due to the sustained growth in the application of contemporary AI methods in clinical oncology, particularly in medical imaging analysis, radiomics and predictive modelling, as well as the increased availability of digital clinical data and computational capacity.

The search strategy combined controlled vocabulary terms (MeSH/DeCS) and free-text keywords related to AI, machine learning, deep learning, radiomics, head and neck cancer, diagnosis, risk stratification, lymph node metastasis and extranodal extension, using Boolean operators AND and OR. The complete search strategies adapted to each database are presented in **Table 1**.

**Table 1:** Search strategies.

Database	Search algorithm
PubMed	(“Head and Neck Neoplasms”[Mesh] OR “head and neck cancer*” OR “head and neck carcinoma*” OR “oral cancer*” OR “oropharyngeal cancer*” OR “laryngeal cancer*” OR “hypopharyngeal cancer*”) AND (“Artificial Intelligence”[Mesh] OR “Machine Learning”[Mesh] OR “Deep Learning”[Mesh] OR artificial intelligence OR machine learning OR deep learning OR neural network* OR radiomic* OR algorithm*) AND (diagnos* OR predict* OR prognos* OR “risk stratification” OR classification)
Web of Science	TS=((“head and neck cancer*” OR “head and neck neoplasm*” OR “oral cancer*” OR “oropharyngeal cancer*” OR “laryngeal cancer*”) AND (“artificial intelligence” OR “machine learning” OR “deep learning” OR “neural network*” OR radiomic* OR algorithm*) AND (diagnos* OR predict* OR prognos* OR “risk stratification” OR classification))
Scopus	TITLE-ABS-KEY((“head and neck cancer*” OR “head and neck carcinoma*” OR “oral cancer*” OR “oropharyngeal cancer*” OR “laryngeal cancer*”) AND (“artificial intelligence” OR “machine learning” OR “deep learning” OR “neural network*” OR radiomic* OR algorithm*) AND (diagnos* OR predict* OR prognos* OR “risk stratification” OR classification))
IEEE Xplore	(“head and neck cancer” OR “oral cancer” OR “oropharyngeal cancer” OR “laryngeal cancer”) AND (“artificial intelligence” OR “machine learning” OR “deep learning” OR “neural network” OR radiomics)

In addition, a manual search was performed by reviewing the reference lists of studies assessed at the full-text stage (snowballing). All retrieved records were managed using the Rayyan platform, which enabled structured reference organization and duplicate removal.

### Selection of studies

Following duplicate removal, unique records underwent title and abstract screening conducted independently by two reviewers. Potentially eligible studies were subsequently assessed at full text to verify alignment with the predefined PCC framework.

During full-text assessment, studies were excluded if they did not address clinical applications of AI, did not include populations with head and neck cancer, or failed to provide relevant information for diagnostic or risk stratification tasks. Disagreements between reviewers were resolved by consensus and, when necessary, through the involvement of a third reviewer.

The complete study selection process is summarized in the PRISMA-ScR flow diagram.

### Inclusion and exclusion criteria

Studies published between January 2015 and January 2026, in English or Spanish, were included if they explicitly evaluated the application of AI methods for the diagnosis or risk stratification of head and neck cancer in adult populations within real-world clinical settings.

Eligible study designs included prospective or retrospective observational studies, cohort studies, investigations focused on the development and validation of machine learning, radiomics or deep learning models, as well as systematic reviews and umbrella reviews that contributed relevant information to characterize the state of knowledge in this emerging field.

Studies were required to use real clinical data, including medical imaging (CT, MRI, PET/CT), digital histopathological images or multimodal approaches integrating clinical, radiological or pathological information, and to evaluate clinically relevant outcomes related to diagnosis or risk stratification, such as lymph node metastasis, extranodal extension, tumor recurrence, survival or treatment response.

Studies were excluded if they did not employ AI techniques or were limited to conventional statistical models without an algorithmic component; if they were preclinical or animal studies; simulations without real clinical data; studies conducted exclusively in pediatric populations; investigations focused on cancers other than head and neck cancer; or non-original publications lacking an empirical basis (editorials, letters to the editor, opinion pieces, protocols without results, conference abstracts without full text and individual case reports).

### Data extraction

Data extraction was performed using a structured matrix developed in Microsoft Excel, specifically designed for this review and aligned with the study objectives.

Extracted variables included: title, authors, year of publication, country, source database, study design, clinical population, care setting, sample size, primary clinical objective, type of AI model, data source and modality (CT, MRI, PET/CT, radiomics, digital histopathology), predicted outcomes, performance metrics, validation strategies and the main methodological limitations reported.

Data extraction was conducted independently by two reviewers, with discrepancies resolved through consensus.

### Data analysis and synthesis

In line with the objectives of a scoping review, data analysis was conducted using a descriptive and narrative approach. Synthesis tables were developed to organize and characterize the main clinical and methodological features of the included studies, including the types of AI models employed, data modalities used and clinical outcomes addressed.

Studies were grouped according to the type of clinical task (diagnosis, risk stratification or both), data modality (radiomics, radiological imaging, digital histopathology or multimodal approaches) and algorithm type (machine learning, deep learning or other hybrid approaches). This classification enabled identification of recurrent patterns, methodological variability and emerging trends in the application of AI to head and neck cancer.

In accordance with Joanna Briggs Institute recommendations for scoping reviews, no formal assessment of methodological quality or risk of bias was performed, as the primary objective was to map and characterize the available evidence. Nevertheless, the main methodological limitations reported by study authors were descriptively considered to contextualize the findings and inform future research directions.

## Results

### Study selection

The bibliographic search identified a substantial body of recent literature on AI applied to head and neck cancer, reflecting the rapid growth of the field. In total, 657 records were retrieved from PubMed, Scopus, Web of Science and IEEE Xplore. After removal of 17 duplicates, 648 unique records were screened by title and abstract.

**Figure 1:**
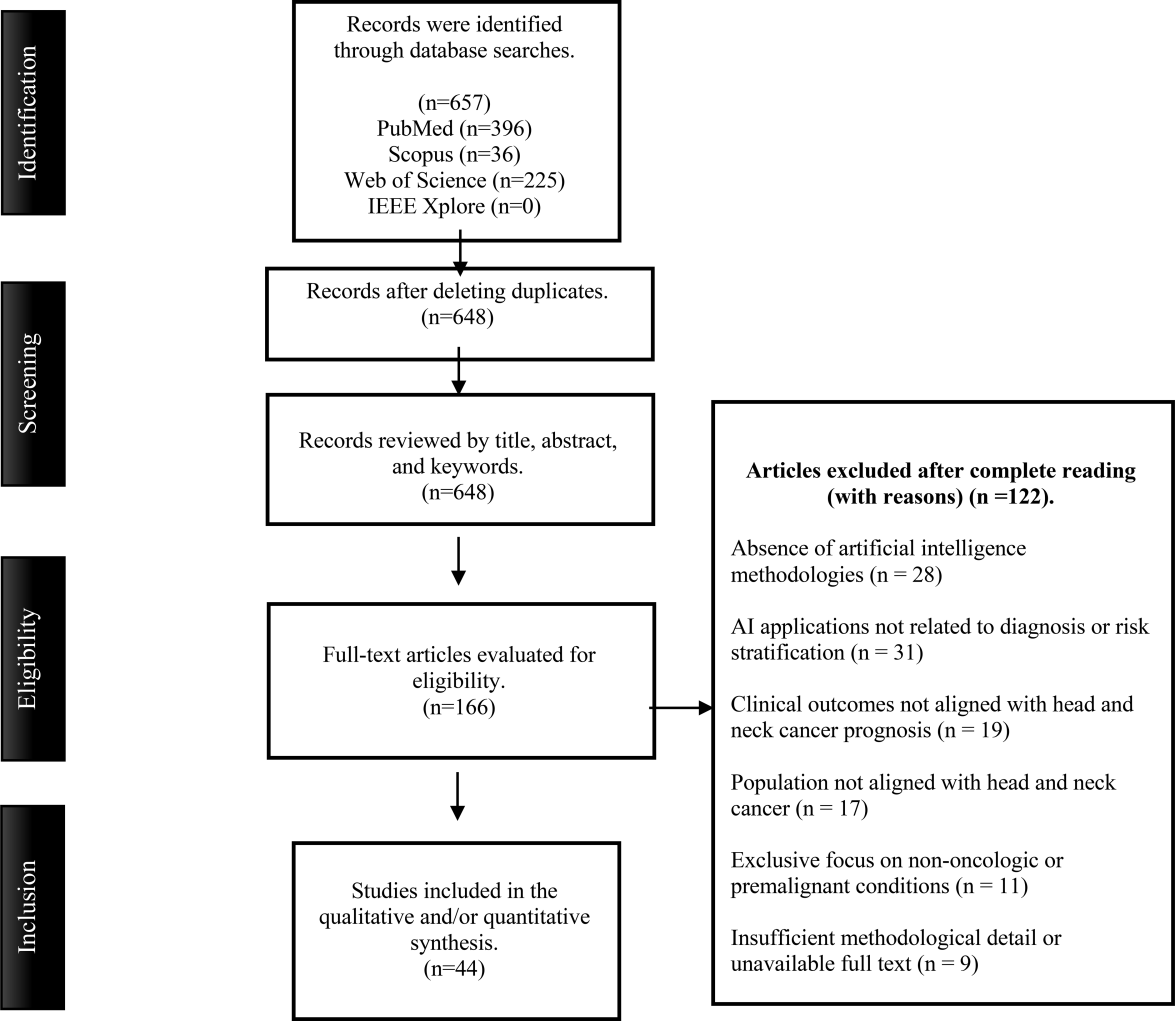
PRISMA-ScR flow diagram.

At this initial stage, studies were excluded if they did not address head and neck cancer, did not employ AI methodologies, or were limited to traditional statistical analyses without an algorithmic component. Subsequently, 166 articles were assessed at the full-text level; of these, 122 were excluded for not addressing clinical objectives related to diagnosis or risk stratification. Ultimately, 44 studies met the eligibility criteria and were included in the qualitative synthesis. The complete study selection process is summarized in the PRISMA-ScR flow diagram (**Figure 1**).

Overall, this process demonstrates a progressive filtering towards studies with concrete clinical applications of AI, aligned with diagnostic and risk stratification objectives.

### General characteristics of included studies

The 44 included studies encompassed a broad range of AI applications in the diagnosis and risk stratification of head and neck cancer, reflecting the notable clinical, anatomical and methodological diversity of the field. The studies addressed nasopharyngeal, laryngeal, oropharyngeal and oral cavity tumors, as well as mixed cohorts of head and neck squamous cell carcinoma (1–44).

The investigations revealed substantial diversity in terms of patient populations, clinical objectives, data types, algorithmic approaches and validation methods. **Table 2** describes these characteristics in a detailed and individualized manner, enabling direct comparison across studies and summarizing the most relevant methodological and clinical elements of each included investigation.

**Table 2:** Predictive application domains of artificial intelligence in head and neck cancer risk stratification.

AI application domain	Primary clinical task	Dominant data modalities	Typical AI approaches	Overall performance trend	Main limitations identified	Key references
Tumour diagnosis and classification	Detection/differentiation of malignant vs premalignant lesions; tumour subtype classification	Histopathology (WSI), CT, clinical images	CNNs, deep learning classifiers, ML ensembles	Moderate–high diagnostic accuracy	Retrospective designs; limited external validation; variable image acquisition	(6–8, 20, 40–42)
Lymph node metastasis prediction	Detection of occult nodal disease	CT, MRI, PET/CT radiomics	Radiomics + ML (RF, SVM); DL	Moderate–high AUC	Heterogeneous segmentation pipelines; monocentric cohorts	(1, 2, 9, 16, 30, 33, 36)
Extranodal extension prediction	Preoperative risk stratification	CT, MRI radiomics	Radiomics-based ML; DL	Moderate–high discrimination	Lack of standardised outcome definitions; limited prospective validation	(1, 2, 16, 30)
Prognostic risk stratification	Prediction of recurrence, OS, DFS, PFS	CT, PET/CT, radiomics, clinical data	ML survival models; DL radiomics	Moderate–high prognostic performance	Small sample sizes; overfitting risk; limited calibration analysis	(3, 4, 11, 13, 15, 18, 26, 27, 31)
HPV status prediction	Molecular surrogate classification	CT radiomics, imaging features	Radiomics + ML	Moderate–high accuracy	Variable imaging protocols; limited clinical integration	(14, 26, 29)
Multimodal risk modelling	Integrated precision stratification	Radiology + histopathology + clinical data	Multimodal DL, hybrid models	Potential performance improvement	Data harmonisation challenges; computational complexity	(30, 33–35, 37)
Clinical decision support (exploratory)	Treatment guidance and patient selection	Clinical variables + imaging	ML-based CDSS; DL	Exploratory feasibility	Lack of prospective impact assessment	(39–41)

To provide a synthetic overview of predominant patterns in the literature and to complement this detailed description, **Table 3** presents a cross-sectional summary of clinical AI applications in head and neck cancer. This summary is structured according to the type of clinical task addressed, data modality employed, class of AI model and level of validation achieved. This table allows precise identification of recurring trends, such as the widespread application of radiomics and deep learning models to radiological imaging and the limited external validation observed across most proposed approaches.

**Table 3:** Synthesis of clinical AI applications in head and neck cancer.

Type of AI application	Data modality	AI model type	Level of validation	Main limitations
Diagnosis and tumour classification	CT, MRI, Histopathology WSI	DL, ML, Radiomics	Mostly internal	Retrospective designs, limited external validation
Lymph node metastasis prediction	CT, MRI, PET/CT	Radiomics, ML, DL	Internal; limited external	Heterogeneous imaging protocols, monocentric cohorts
Extranodal extension prediction	CT, MRI	Radiomics, ML	Internal; few external	Small sample sizes, segmentation variability
Prognostic stratification (recurrence, survival)	CT, MRI, PET/CT, Clinical data	Radiomics, ML, DL	Predominantly internal	Outcome heterogeneity, lack of prospective validation
Therapeutic decision support (HPV status, treatment intensity)	MRI, PET/CT, Clinical + imaging	DL, Multimodal ML	Internal	Limited clinical impact evaluation
Multimodal risk stratification	Imaging + Histopathology + Clinical	Hybrid ML/DL	Internal; rare external	Data integration challenges, computational complexity

Overall, the evidence is methodologically diverse but converges on the use of AI as an emerging tool to support diagnostic and risk stratification processes in head and neck oncology.

### Geographical distribution

From a geographical perspective, the evidence was concentrated primarily in high- and middle-income countries, with predominant representation from Japan, the United States, Western Europe and China (1–6, 9–13, 16–21, 27–30).

A limited number of studies employed multicentre designs or international collaborations (2, 3, 16, 17, 33); however, most investigations were based on single-institution cohorts, which constrains the generalizability of the findings.

**Figure 2:**
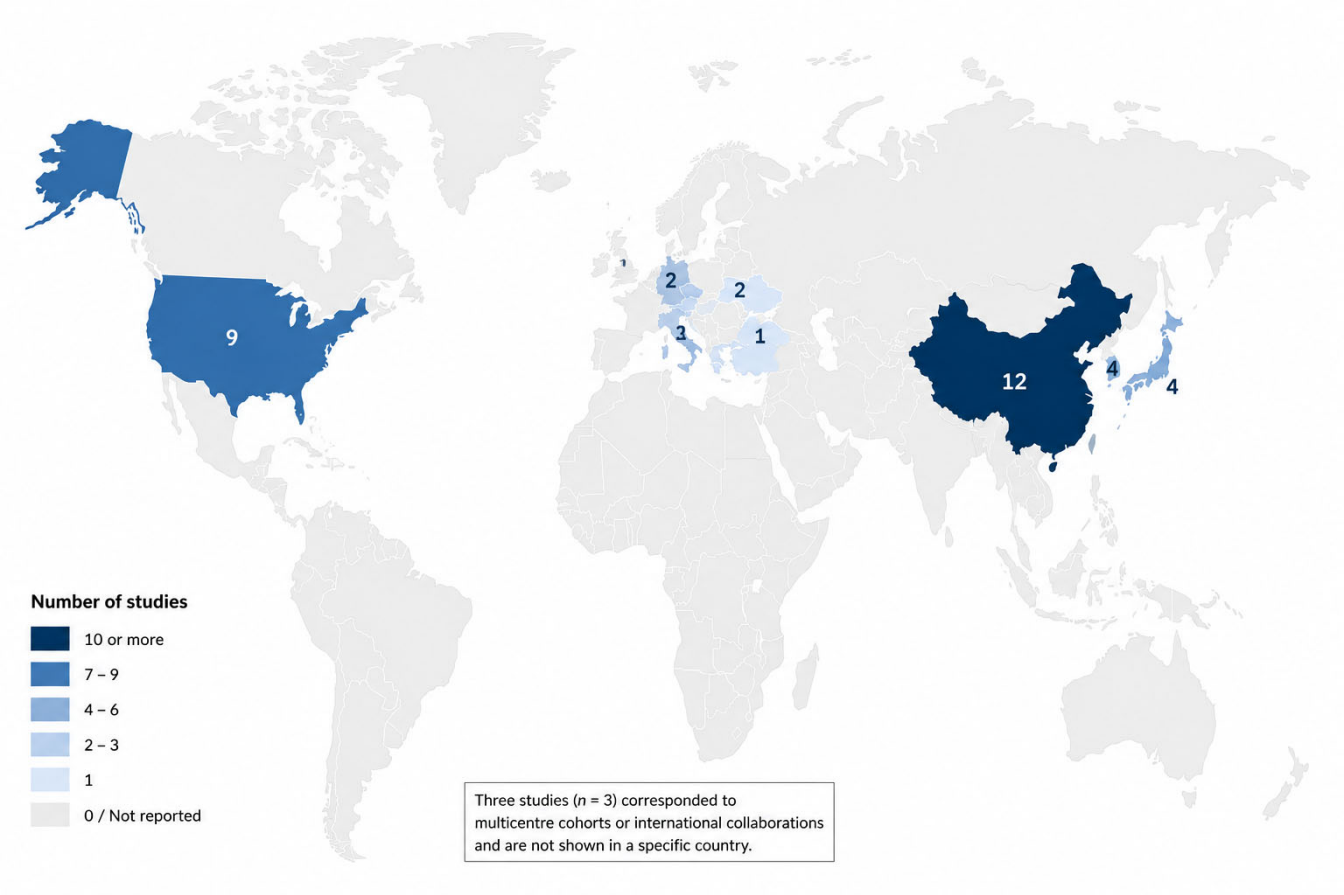
Geographical distribution of included studies. **Figure 2** illustrates the geographical distribution of the 44 included studies. China was the country with the highest number of publications (n = 12), followed by the United States (n = 9) and Japan (n = 6). South Korea contributed four studies (n = 4). In Europe, Italy (n = 3), Germany (n = 2), the Netherlands (n = 2), Poland (n = 2) and Romania (n = 1) were represented. In addition, three studies (n = 3) corresponded to multicentre cohorts or international collaborations. Map created using Natural Earth (https://www.naturalearthdata.com/about/terms-of-use/).

Head and neck cancer exhibits the largest global frequency in Asia, especially in the Melanesia and South Central Asia subregions, where incidence and fatality rates are significantly raised (**Figure 2**). GLOBOCAN data indicates that the highest age-standardized incidence rates of lip and oral cavity cancer globally are found in Melanesia, South Central Asia, and Southeast Asia, regions where tobacco smoking, smokeless tobacco use, betel quid chewing, and areca nut consumption are prevalent risk factors affecting the population.

Laryngeal and hypopharyngeal cancers impose a significant burden in South Asia and certain regions of Southeast Asia, frequently identified at advanced stages owing to restricted access to early detection services and specialized oncology care. Conversely, AI-focused research from these high-burden areas is significantly under-represented in the existing literature. This significant geographic disparity is not just a bibliometric anomaly but a crucial scientific and ethical issue: if AI models are consistently developed and validated in populations from East Asia, Western Europe, and North America, their biological, anatomical, demographic, and imaging-protocol assumptions may not generalize to the populations most affected by disease.

The structural impediments contributing to this disparity are multifaceted. Challenges encompass restricted institutional access to extensive annotated imaging datasets, inadequate computational infrastructure, limited research funding in low- and middle-income regions, language obstacles in scientific publications, and the concentration of academic oncology expertise in tertiary centers primarily situated in high-income urban areas. The prevalent reliance on CT and MRI as data modalities in published AI studies presupposes imaging accessibility that is not universally available in South Asia or the Pacific, where plain radiography or clinical examination may serve as the primary diagnostic resource at the point of care. In light of this acknowledged deficiency, the search method for this review was broadened to explicitly target AI-related articles from Asian institutions and regional databases. This comprehensive search revealed numerous pertinent contributions.

Nasopharyngeal carcinoma (NPC), a cancer subtype with significantly increased prevalence in Southern China, Southeast Asia, and North Africa, is strongly linked to Epstein-Barr virus infection and has been the focus of numerous high-quality AI studies conducted by Chinese institutes. Zhang et al. created an MRI-based deep learning model to predict distant metastasis-free survival in a multicenter Chinese cohort (34).

Chen et al. developed a deep learning radiomic nomogram that integrates MRI characteristics and clinical data from 1,872 patients across four Chinese centers to inform treatment decisions in locoregionally progressed NPC, with concordance indices reported between 0.784 and 0.921 (35). Li et al. established that integrating peritumoral regions into deep learning models using MRI enhanced prognosis prediction in nasopharyngeal carcinoma beyond tumor-only methodologies, indicating that the microenvironment surrounding the primary tumor harbors prognostically significant information overlooked by traditional tumor delineation.

Yi et al. created a multimodal radiopathomics model that combines pretreatment MRI and whole slide images to predict overall survival in locally advanced NPC, with external validation conducted at a Chinese cancer center, demonstrating the efficacy of multimodal data fusion in high-burden endemic populations (37). Khongwirotphan et al. from Thailand utilized multimodal radiomics incorporating PET/CT and MRI for the prognosis of nasopharyngeal carcinoma (NPC), marking one of the rare contributions of artificial intelligence from Southeast Asia, a region with a clinically significant incidence of NPC (38).

Yang et al. examined AI applications for the diagnosis and treatment of NPC from a Chinese viewpoint, offering a thorough synthesis of the current advancements in endemic areas where NPC represents a significant public health concern (39). Hegde et al. examined AI applications for the early diagnosis of oral cancer, focusing on the Asia-Pacific region. They emphasized the critical necessity for AI tools tailored to the unique epidemiological and etiological characteristics of oral cancer in this area, where betel nut consumption is the primary risk factor, in contrast to HPV infection prevalent in Western nations (40).

Lan et al. from Sun Yat-sen University devised an MRI-based deep learning and radiomics model to predict occult cervical lymph node metastasis in early-stage oral and oropharyngeal squamous cell carcinoma, utilizing data from 319 patients across various Chinese centers, and demonstrating high diagnostic accuracy with AUC values significantly surpassing traditional clinical evaluation (41). Wang et al. executed a pivotal multicenter study involving four Chinese institutions to compare 3D and 2D deep learning, radiomics, and fusion models for predicting occult lymph node metastasis in laryngeal squamous cell carcinoma, utilizing CT imaging from 553 patients. The study revealed that decision-level fusion models incorporating multiple data streams exhibited enhanced predictive performance (42).

Liang et al. introduced a deep learning radiomics nomogram for predicting lymph node metastasis in laryngeal squamous cell carcinoma, enhancing the accumulating evidence for the therapeutic applicability of AI in this histological subtype (43). Jiang et al. established and validated a CT-based deep learning radiomics signature for predicting lymph node metastasis in oropharyngeal squamous cell carcinoma through a multicenter Chinese study, revealing that the integration of deep learning features with traditional radiomic descriptors enhanced predictive accuracy beyond the efficacy of either method independently (44). The collective contributions from Asian institutions indicate that high-quality AI research in head and neck cancer is achievable in the region, and that endemic tumor subtypes, such as NPC and betel-quid-associated oral cancer, warrant focused methodological consideration.

Nonetheless, research from high-burden regions like India, Bangladesh, Pakistan, Papua New Guinea, and the Pacific Islands is notably lacking in the AI literature, despite these areas collectively representing a disproportionate share of global head and neck cancer incidence and mortality. India ranks among the nations with the highest absolute incidence of oral cavity cancer globally, and the lack of AI-based risk stratification studies from Indian academic institutions constitutes a significant gap that necessitates immediate attention. This under-representation may indicate not only structural and physical obstacles but also systematic imbalances in the priority of research funding, access to prestigious journals, and the valuation of locally-produced data within global scientific dialogue.

To address this gap, intentional and ongoing efforts are necessary at various levels: research funders must incentivize AI studies in high-burden, low-resource environments; journal editors should implement submission policies that mitigate language and formatting obstacles; and international collaborations ought to be designed to enhance local AI capabilities instead of solely utilizing local patient data for models that are managed externally. Systematic reviews in this domain should broaden their search strategies to encompass regional Asian databases such as IndMED, KoreaMed, the Western Pacific Index Medicus, and the Index Medicus for the South-East Asia Region (IMSEAR), along with non-English language publications and grey literature, to guarantee a comprehensive, inclusive, and equitable representation of evidence.

**Figure 2** illustrates this geographical distribution and highlights the underrepresentation of low-income regions, raising important questions regarding equity, transferability and the global applicability of the developed models.

### Study design and populations

In terms of study design, retrospective observational research predominated, based on clinical databases, institutional medical imaging repositories and digital pathology archives (3, 9–15, 18–21, 23–30, 33–36).

A relevant subset consisted of systematic reviews and meta-analyses synthesizing existing evidence on AI applications in oral cancer and head and neck cancer (1, 2, 6, 7, 20, 21). These reviews contributed to contextualizing both technical advances and methodological limitations within the field.

Most studies included adult patients with histologically confirmed squamous cell carcinoma, although with variability in tumor subsite, clinical stage and therapeutic context (4, 9, 14, 18, 25, 27, 30). This clinical heterogeneity further underscores the complexity of developing generalizable predictive models. **Table 4** presents a comparative summary of included studies, datasets, AI modalities, validation strategies and performance metrics.

**Table 4:** Comparative summary of included studies: datasets, modality, prediction target, validation type and performance metrics.

Study (first author, year)	Cancer subsite	Data modality	AI approach	Prediction target	Validation type	AUC/Key metric	Sample size
Stawarz et al., 2025 (1)	Oropharyngeal	CT, MRI	Radiomics + ML	ENE, LNM	Systematic review	Pooled AUC varied	Multiple studies
Valizadeh et al., 2025 (2)	Head & neck (mixed)	CT, MRI, PET/CT	Radiomics, DL, ML	LNM	Meta-analysis (internal + external)	AUC 0.73–0.97	Multiple studies
Shannon et al., 2025 (4)	Nasopharyngeal	CT, MRI	Radiomics + DL	Prognosis, recurrence	Internal	AUC >0.75	Moderate
Huynh et al., 2023 (3)	Head & neck (mixed)	CT	ML (radiomics) vs DL radiomics	Treatment outcome	Internal CV	Comparable AUC	~200
Jiang et al., 2023	Oral cavity	CT	Radiomics + ML	Occult LNM	Internal	AUC 0.83	Moderate
Michelutti et al., 2023 (10)	Head & neck (mixed)	CT, MRI	ML, DL algorithms	Prognosis, LNM	Internal	AUC 0.75–0.90	Moderate
Li et al., 2023 (36)	HNSCC	CT	AI-based	ENE prediction	Internal	AUC 0.80+	Moderate
Araújo et al., 2025 (14)	Oropharyngeal	CT radiomics	Radiomics + ML	HPV status	Internal	AUC >0.80	Moderate
Philip et al., 2023 (15)	HNSCC	PET/CT	Radiomics	Outcome prediction	Internal	Moderate–high	Moderate
Chen et al., 2024 (24)	Head & neck (mixed)	MRI + clinical	Multimodal DL	Prognosis	Internal	Moderate–high	Moderate
Wang et al., 2024 (23)	Laryngeal	CT	Radiomics (interpretable)	Classification	Internal	High accuracy	Moderate
Song et al., 2025 (33)	Oropharyngeal	CT	Deep learning (primary tumour + LN)	Outcome prediction	External (multi-institutional)	High performance	Large
Bogowicz et al., 2019 (11)	Head & neck (mixed)	CT	DL vs hand-crafted radiomics	Recurrence	Internal	Moderate	~200
Lou et al., 2019 (12)	Head & neck (mixed)	CT	Radiomics	Local control	Internal	AUC ~0.70	~100
Elhalawani et al., 2018 (18)	Head & neck (mixed)	CT, MRI	ML (multiple)	RT outcome	Internal	Varied	Large
Diamant et al., 2019 (19)	Head & neck (mixed)	CT	Deep learning	Outcome prediction	Internal	Moderate–high	~200
Zhang et al., 2017 (16)	Nasopharyngeal	MRI	Radiomics + ML	Prognostic biomarkers	External	AUC 0.82	Moderate

### Data modalities and AI approaches

Radiological imaging constituted the predominant data modality, particularly CT, MRI and PET/CT, used both for radiomic feature extraction and for training predictive models (3, 4, 9–15, 18, 23–30, 33–36).

A smaller number of studies incorporated digital histopathology through whole-slide imaging, structured clinical variables or multimodal approaches integrating multiple data sources (5, 17, 19, 29, 35, 37).

From a methodological standpoint, two dominant approaches were identified:

1. Classical machine learning, including Random Forest, Support Vector Machines and ensemble models (3, 9, 12, 18, 23, 24, 27, 30, 33).
2. Deep learning, primarily convolutional neural networks applied to medical or histopathological images, in some cases combined with radiomics (4, 10, 14, 17, 25, 28, 35).

Collectively, these findings indicate a progressive transition from traditional models towards more complex architectures capable of capturing high-dimensional information.

### Clinical objectives and predicted outcomes

The clinical objectives addressed by AI models were grouped into four main domains, enabling a structured understanding of their clinical utility:

1. Tumor diagnosis and classification, including detection of oral cancer, dysplasia and differentiation of tumor subtypes (6–8, 20, 40–42).
2. Prediction of lymph node metastasis and extranodal extension, particularly in oral cavity and oropharyngeal cancers (1, 2, 9, 16, 30, 33, 36).
3. Prognostic stratification, aimed at predicting recurrence, local control, disease-free survival and overall survival (3, 4, 11, 13, 15, 18, 26, 27).
4. Therapeutic decision support, such as prediction of HPV status or identification of patients suitable for treatment intensification or de-escalation (14, 26, 29).

Reported performance metrics were heterogeneous; however, most studies employed AUC, accuracy, sensitivity and specificity, with generally moderate to high performance, albeit with variability across studies (1–10, 44).

### Validation strategies and methodological limitations

Regarding validation, internal validation predominated, using cross-validation techniques or training–testing splits (3, 9, 12, 18, 23, 27, 33). External validation was less frequent and, when performed, was limited to small independent cohorts (2, 16, 33).

Consistently, studies shared recurrent methodological limitations, including the absence of systematic calibration and interpretability analyses, variability in segmentation and feature extraction processes, a predominance of single-center designs and moderate sample sizes (1–7, 20, 21, 30, 33–36). Taken together, these findings indicate that although the models demonstrate promising performance, significant barriers remain to robust external validation and large-scale clinical implementation.

## Discussion

### Artificial intelligence as a methodological response to unmet clinical needs in head and neck oncology

Head and neck cancer continues to represent one of the most complex oncological scenarios from a clinical perspective, owing to its pronounced anatomical, histopathological and biological heterogeneity. The diversity of tumor subsites, the variable influence of human papillomavirus infection, and the considerable inter-individual variability in treatment response collectively limit the ability of conventional staging systems, including TNM, to accurately estimate individual patient risk (1–4). Although the TNM system and the incorporation of etiological factors such as HPV status have improved prognostic stratification, relevant discrepancies persist between initial classification and actual tumor behavior, particularly with regard to nodal dissemination, recurrence and survival (15–18). These limitations have driven the search for more sophisticated analytical approaches capable of integrating multiple dimensions of clinical and biological information simultaneously.

In this context, the studies included in this review demonstrate that AI emerges as a direct methodological response to these unmet clinical needs by enabling the simultaneous analysis of complex, high-dimensional and non-linear data. Machine learning and deep learning models have shown the ability to identify predictive patterns that extend beyond conventional clinical variables, extracting information from imaging, pathology and clinical data that is not accessible to standard visual assessment or statistical regression (6–8). These models provide more individualized risk estimates that may be better aligned with real tumor behavior, potentially enabling more precise patient selection for treatment intensification, de-escalation or clinical trial enrolment (19–22).

### Radiomics-driven risk stratification: strengths and reproducibility challenges

Radiomics is the predominant methodology employed in AI applications for head and neck cancer, especially for predicting lymph node metastases, extranodal extension, and prognostic outcomes. Research utilizing CT, MRI, and PET/CT has demonstrated that quantitative radiomic features encapsulate elements of intratumoral heterogeneity, spatial texture, and morphological irregularity linked to unfavorable clinical outcomes, such as locoregional recurrence, diminished disease-free survival, and overall survival. The radiomic methodology has been utilized for nasopharyngeal, laryngeal, oropharyngeal, and oral cavity tumors, with the majority of research indicating moderate to high discriminative efficacy as assessed by AUC, hence exhibiting a consistent enhancement beyond clinical factors alone (34, 36, 38, 45, 47).

This review emphasizes that the theoretical robustness of radiomics is undermined by substantial repeatability issues, which restrict its present practical utility. Variations in image acquisition protocols, reconstruction kernels, tumor segmentation techniques, feature selection methods, and validation strategies create significant methodological heterogeneity among studies, rendering direct result comparisons unreliable and undermining the external validity of reported performance metrics (24, 26, 28). The lack of standardized radiomic feature extraction protocols and the absence of prospective multicenter validation are the primary obstacles to the integration of radiomics-based models into standard oncological practice, highlighting the necessity for consensus-driven harmonization initiatives (42, 44, 47).

### Deep learning in head and neck cancer: representational power versus clinical transparency

Deep learning models, especially convolutional neural networks utilized for medical and histopathological images, signify a significant methodological advancement by facilitating the automatic acquisition of hierarchical representations directly from raw pixel data, eliminating the need for manual feature engineering. These methodologies have exhibited encouraging efficacy across various tasks, including tumor detection, malignancy grading, lymph node status prediction, and survival estimation, with performance metrics often comparable to or surpassing expert radiological or pathological evaluations in controlled experimental conditions (29, 30, 34–37, 41, 43).

However, the analysis suggests that this enhancement in predictive capability frequently correlates with diminished clinical interpretability, resulting in a fundamental conflict between model efficacy and clinical applicability. The opaque nature of numerous deep learning architectures complicates clinicians’ ability to comprehend, validate, or challenge the determinants of individual predictions, which is especially crucial in head and neck cancer, where treatment decisions may lead to substantial and irreversible functional consequences impacting speech, swallowing, and respiration (5, 22, 29). Moreover, the majority of these models were constructed utilizing retrospective single-center cohorts with moderate sample sizes, thus heightening the danger of overfitting, constraining generalizability, and producing optimistically skewed performance estimates (36, 43, 45, 47).

### Multimodal artificial intelligence and the transition toward precision oncology

Numerous studies have progressed toward multimodal AI models that incorporate radiographic, histological, and clinical data, aligning with the conceptual tenets of precision oncology, which acknowledge cancer as a multifaceted biological phenomenon inadequately represented by any singular data modality. These methodologies indicate that integrating imaging characteristics with molecular, genomic, or structured clinical variables may augment risk stratification, facilitate the identification of biologically distinct patient subgroups, and allow for more refined treatment personalization compared to unimodal models (30, 33–35, 37). Multimodal designs that incorporate MRI radiomics, whole slide imaging, and clinical staging have shown particularly encouraging outcomes in nasopharyngeal carcinoma and oral cavity malignancies (37, 41, 46).

Nonetheless, the implementation of multimodal AI models in clinical practice encounters significant practical obstacles that presently restrict their extensive acceptance outside of academic environments. These encompass the incomplete availability of all necessary data modalities across institutions, significant difficulties in harmonizing diverse data sources with varying formats, acquisition protocols, and quality standards, as well as a pronounced increase in computational complexity and infrastructure demands compared to unimodal approaches (13, 14). Furthermore, limited research has systematically assessed the incremental prognostic advantage provided by each individual modality, complicating the identification of which data streams genuinely enhance predictive accuracy and which merely introduce noise or redundancy, thus constraining the clinical interpretation of the actual added value of multimodal integration (36, 38, 41, 46).

### Clinical implications: decision support in a high-morbidity setting

From a clinical standpoint, the research repeatedly highlight that AI should be seen as a clinical decision support tool that enhances, rather than supplants, human judgement. In head and neck cancer, where treatment choices significantly affect functional outcomes such as speech, swallowing, airway protection, and quality of life, AI serves to enhance the clinician’s evaluation by offering quantitative, imaging-based risk assessments that would otherwise remain unattainable. The evaluated models exhibit the ability to forecast nodal metastases, extranodal extension, HPV status, and survival outcomes with clinically significant precision, providing a foundation for more personalized treatment strategies and patient guidance in multidisciplinary tumor boards (39, 41–44).

A crucial differentiation must be made between research-level algorithmic efficacy and authentic clinical preparedness for practical implementation, a distinction that holds significant ramifications for the interpretation and use of the evidence examined herein. The bulk of research reported performance metrics based on retrospective, single-center cohorts utilizing internal cross-validation or fixed training-test splits, settings that are systematically more advantageous than those faced in clinical practice (3, 9, 11, 12). Retrospective datasets are generally more refined, comprehensive, and internally consistent than prospectively gathered real-world data; models validated internally utilize identical acquisition protocols, scanner specifications, and patient demographics as their training sets; furthermore, the lack of temporal separation implies that models are not evaluated against the genuine challenge of forecasting outcomes for untreated patients.

These conditions fundamentally contrast with prospective clinical deployment, where models must consistently perform across patient populations with varying comorbidity profiles, diverse imaging equipment from multiple manufacturers and hardware generations, evolving institutional workflows, and real-time data entry by clinical personnel who may not follow standardized documentation practices (22, 30). A model attaining an AUC of 0.90 in an internal validation set from a singular academic institution may significantly deteriorate when implemented in a community hospital with divergent CT acquisition protocols, varying tumor delineation standards, or a patient demographic characterized by a higher prevalence of advanced-stage disease or increased comorbidity burden (42, 44, 47). External validation in independent, prospective, multicenter cohorts from geographically and institutionally diverse locations is the gold standard for establishing clinical readiness; nonetheless, it was observed in just a minority of the included studies (1, 2, 16, 33).

The clinical implications of AI prediction errors are notably significant in head and neck oncology, as model outputs may directly influence decisions regarding the extent of surgical resection, elective neck dissection, the intensification or de-escalation of chemoradiotherapy, and patient eligibility for clinical trials (20, 22, 39). An AI model that inaccurately assesses nodal metastasis risk may subject a patient to unwarranted neck dissection, resulting in potential complications such as chronic lymphoedema, shoulder dysfunction, and cranial nerve injury; conversely, an underestimation of extranodal extension risk may result in insufficient adjuvant therapy and subsequent locoregional recurrence (1, 2, 9, 30). No included studies prospectively evaluated the utilization of AI-derived predictions by clinicians, their influence on management decisions, or their subsequent effects on patient outcomes and quality of life, highlighting the most significant evidence gap identified in this review (39–41, 46).

In addition to technical efficacy, authentic clinical readiness necessitates the seamless integration of AI tools into electronic health record systems with minimal disruption to clinical workflows; predictions must be delivered in interpretable, actionable formats rather than as obscure probability scores that clinicians cannot effectively analyze or challenge; and end-users must receive sufficient training to comprehend model outputs, their confidence intervals, and their recognized failure modes (5, 13, 14, 22). The regulatory frameworks for AI-driven clinical decision support systems in oncology are underdeveloped in many regions, and the lack of defined approval criteria generates ambiguity regarding the appropriate timing and methods for their lawful use beyond restricted research environments (44, 46). Future research should emphasize prospective trials that assess AI tools in genuine clinical settings, including patient-centered outcomes such as functional preservation, quality of life, and the shared decision-making experience (39–41, 46).

### Methodological rigor, external validity, and governance

The methodological shortcomings observed in the included research are consistent and pervasive, highlighting greater issues in the creation of clinically relevant AI models in oncology. Retrospective designs, moderate sample sizes, and dependence on single-center cohorts introduce risks of optimistic bias, constrain statistical power for subgroup analyses, and limit the external validity of suggested models to the specific institutional environment in which they were developed (23, 25, 30, 33, 42, 44, 47). The prevalence of internal validation strategies, typically utilized on datasets comprising fewer than 200 patients, significantly heightens the likelihood that reported performance metrics exaggerate actual generalizable accuracy, especially for deep learning models with extensive trainable parameters (11, 12, 43).

Additionally, the significant variability in the definition and operationalization of clinical outcomes across studies impedes meaningful comparison, obstructs quantitative meta-analytic synthesis, and restricts the applicability of findings to standardized clinical decision frameworks (42, 43, 45). Progressing towards more resilient algorithmic governance is consequently a strategic imperative for the domain, involving obligatory external validation in independent multicenter cohorts, compliance with transparent reporting standards such as TRIPOD-AI, systematic calibration analyses to evaluate concordance between predicted and actual probabilities, interpretability evaluations utilizing tools like SHAP or LIME, and well-defined frameworks for clinical accountability and post-deployment performance oversight.

### Equity, ethics, and future directions

The concentration of AI research in high- and upper-middle-income nations, mostly represented by China, the United States, Japan, and Western Europe, presents significant and unsolved concerns about equity, diversity, and the worldwide applicability of created models. Models developed solely on populations from affluent academic institutions in these areas carry a significant risk of inadequate performance in low- and middle-income contexts, potentially worsening existing disparities in the quality and accessibility of oncological care upon widespread implementation. The recorded under-representation of South Asia, Sub-Saharan Africa, Melanesia, and the Pacific Islands is especially alarming, as these regions bear some of the greatest worldwide burdens of head and neck cancer incidence and mortality.

A mere minority of the studies examined explicitly addressed the ethical and regulatory aspects of AI implementation in oncology, including algorithmic transparency, accountability frameworks for predictive inaccuracies, patient consent for utilizing clinical data in model training, and the incorporation of AI tools into shared decision-making processes that honor patient autonomy and values (41, 44, 46). These problems must be elevated from secondary concerns to strategic research goals, acknowledging that the societal acceptance and equitable implementation of AI in cancer relies equally on governance, ethics, and trust as on technical performance indicators (13, 14, 22). The evidence indicates that advancements in the area will rely less on incremental AUC enhancements and more on a purposeful shift towards translational, ethically grounded, and therapeutically integrated research that clearly benefits patients in all contexts (32, 34, 37, 41, 42, 46).

## Conclusions

This scoping review confirms that AI represents an emerging tool with substantial potential to improve diagnosis and risk stratification in head and neck cancer. The available evidence demonstrates that models based on machine learning, deep learning and radiomics can identify complex tumor patterns associated with lymph node metastasis, extranodal extension, recurrence and adverse oncological outcomes, providing complementary information to conventional clinical and radiological methods.

Nevertheless, the current body of evidence remains predominantly exploratory. Substantial limitations persist, including the widespread use of retrospective designs, single-center cohorts, methodological heterogeneity and limited external validation, all of which restrict the generalizability of findings and their direct clinical adoption. Moreover, a significant gap remains between reported algorithmic performance and the assessment of real-world clinical impact on decision-making, functional outcomes and patient quality of life.

Looking ahead, it is essential to advance towards prospective, multicentre studies with standardized data modalities, consistent definitions of clinical outcomes and robust validation strategies. In addition, the responsible integration of AI into head and neck oncology requires the explicit incorporation of principles of interpretability, algorithmic governance and equity, ensuring that these technologies function as clinical decision support tools rather than substitutes for medical judgement. Taken together, this review provides a structured overview of the current state of knowledge, identifies critical gaps and outlines strategic directions for prudent, ethical and patient-centered clinical implementation.

## Data Availability

The original contributions presented in the study are included in the article. Further inquiries can be directed to the corresponding authors.
